# A review of memristor: material and structure design, device performance, applications and prospects

**DOI:** 10.1080/14686996.2022.2162323

**Published:** 2023-02-28

**Authors:** Yongyue Xiao, Bei Jiang, Zihao Zhang, Shanwu Ke, Yaoyao Jin, Xin Wen, Cong Ye

**Affiliations:** a Hubei Key Laboratory of Ferro-& Piezoelectric Materials and Devices, Faculty of Physics and Electronic Science, Hubei University, Wuhan, China; b Faculty of Chemical Technology and Engineering, West Pomeranian University of Technology in Szczecin, Szczecin, Poland

**Keywords:** Artificial intelligence, in-memory computing, memristor, material and structure design, device performance

## Abstract

With the booming growth of artificial intelligence (AI), the traditional von Neumann computing architecture based on complementary metal oxide semiconductor devices are facing memory wall and power wall. Memristor based in-memory computing can potentially overcome the current bottleneck of computer and achieve hardware breakthrough. In this review, the recent progress of memory devices in material and structure design, device performance and applications are summarized. Various resistive switching materials, including electrodes, binary oxides, perovskites, organics, and two-dimensional materials, are presented and their role in the memristor are discussed. Subsequently, the construction of shaped electrodes, the design of functional layer and other factors influencing the device performance are analyzed. We focus on the modulation of the resistances and the effective methods to enhance the performance. Furthermore, synaptic plasticity, optical-electrical properties, the fashionable applications in logic operation and analog calculation are introduced. Finally, some critical issues such as the resistive switching mechanism, multi-sensory fusion, system-level optimization are discussed.

## GRAPHICAL ABSTRACT

**Figure:**
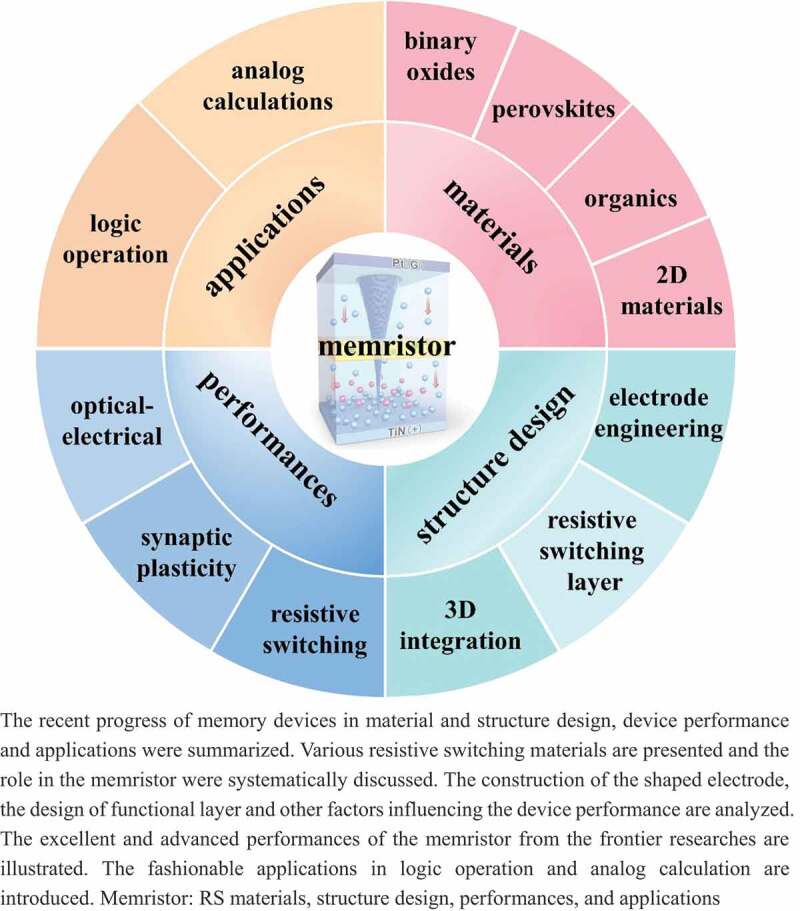

## 1. Introduction

Human society is facing the contradiction between the growing demand for artificial intelligence versus insufficient computing power in the era of big data. The current von Neumann architecture has two serious problems for the separation of storage and computing in intelligent computing [1]: 1. The access speed of memory is far behind the calculation speed of the central processing unit (CPU), and greatly reduces the utilization of the central processing unit, which is called ‘memory wall’ [2,3]; 2. The loss of power consumption caused by data transmission has seriously undermined the development of the chip, which is known as the ‘power wall’ [4]. As predicted by Moore’s law, computing power has been enhanced by continuously reducing device size, alleviating the von Neumann bottleneck [5,6], but these methods still belong to the von Neumann architecture, and the storage and computing of data are still separated from each other, which cannot fundamentally solve the problems. Hence, the researchers investigate new computing systems with non-von Neumann architectures [7,8].

In-memory computing architecture, represented by brain-inspired computing, is one of the non-von Neumann architectures [9,10]. However, its development has been slowed in the past decades, which is mainly due to the lack of a physical device that can realize the architecture of integrated storage and computing system. The problem was not solved until the advent of the memristor. The theoretical memristor was proposed by Leon O. Chua as the fourth basic circuit element in 1971 [11], and the first physical implementation of the memristor was experimentally verified by Strukov et al. at Hewlett-Packard (HP) Labs in 2008 [12]. Memristors have simple structure, fast reading and writing speed, good scalability, high density, low cost, and are compatible with complementary metal oxide semiconductor (CMOS) process.

In the future, memristor is one of the promising candidates for high-density, high-energy efficiency, ultra-fast, low-latency, low power, large-capacity non-volatile memory. Therefore, many companies (Samsung, Panasonic, HP, Micron, Sony, Yangtze Memory Technologies Co., Ltd. (YMTC), Crossbar etc.) are engaged in research and development of memristors. The traditional method to simulate a neuron or a synapse requires dozens of traditional electronic devices such as transistors and capacitors [7]. Although breakthroughs have been made in brain-inspired computing systems based on traditional CMOS technology in recent years [13], there are still many challenges in terms of the integration and power consumption. With a continuously adjustable resistance under an applied electric field, memristors are ideal neurosynaptic bionic components, providing a potential solution for high-density, low-power brain-inspired computing chips. Furthermore, the memristor arrays can build more integrated neural network structures, including artificial neural networks (ANN) convolutional neural networks (CNNs), deep neural networks (DNNs), recurrent neural networks (RNNs), and spike neural networks (SNNs). The memristor arrays directly use Ohm’s law for addition and Kirchhoff’s law for multiplication, thus enabling the parallel multiplication-accumulation (MAC) operations required by neural networks, resulting in a significant increase in the speed and power efficiency of neural networks [14].

**Scheme 1.**
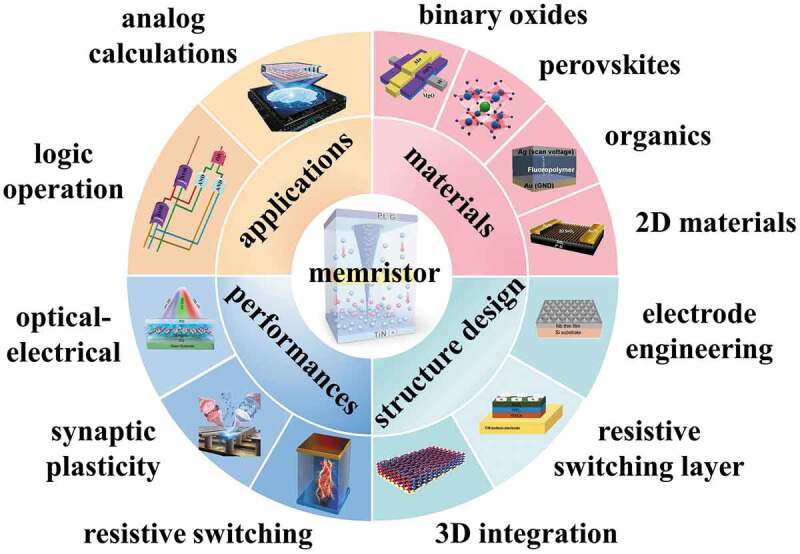
Memristor: RS materials, structure design, performances, and applications. Reproduced with permission from [15,16] copyright 2021, Wiley, [17] copyright 2021, American Chemical Society, [18] copyright 2021, Springer Nature, [19] copyright 2021, American Chemical Society, [20] copyright 2018, Royal Society of Chemistry, [21] copyright 2022, Elsevier, [22] copyright 2020, Springer Nature, [23] copyright 2020, American Chemical Society, [24] copyright 2019, Wiley, [25] copyright 2022, American Chemical Society, [26] copyright 2022, Wiley.

In this review, as shown in Scheme 1, various resistive switching (RS) materials of the memristor are introduced, including common electrodes as well as particular electrode materials, binary oxides, perovskites, organics, and two-dimensional (2D) materials. Next, the latest structural designs, such as electrode engineering, resistive layers, and three-dimensional (3D) integration, are introduced, and their role in the memristor is systematically addressed. Subsequently, the construction of shaped electrodes, the design of functional layers, and other factors affecting device performance are analyzed. Excellent performance of the memristor is illustrated by selected research results. Finally, applications of memristors in logic and simulation are summarized, and some critical issues such as RS mechanisms, multi-sensory fusion, and system-level optimization are looked ahead, providing further developments and new insights for memristor-based in-memory computing.

## 2. Materials of memristors

The memristor mainly has a metal-insulator-metal (MIM) sandwich structure, including two layers of electrodes and a middle resistive layer formed by a semiconductor or insulator. The properties and resistance mechanism of memristors are closely related to the component materials of the devices, which include SET/RESET voltage, on/off ratio, switching speed, endurance, retention, power consumption, etc.

### 2.1 Electrode materials

Memristor electrodes not only carry electric current, but may also participate in the resistive reaction. They are commonly made from the following materials: conventional metals [27–31], noble metals [32,33], alloys [34–36], carbon-based materials such as graphene [37] and carbon nanotubes [38], nitrides such as TiN [16] and TaN [39], transparent conductive flexible oxides such as indium tin oxide (ITO) [29,39,40] and doped ITO [41], F-doped tin oxide (FTO) [42–46], etc. The common electrode materials can be classified into four types according to their different role in RS behavior. First, the electrodes are mainly used as current conducting media and have almost no effect on the RS behavior. Such electrode materials mainly include inert metals, such as Au [32] and Pt [33]. Second, the electrodes are responsible for the formation of conductive filaments (CFs), which is exactly the case of cation migration-based memristors. In these memristors, CFs are formed by electrochemical dissolution and deposition of electrochemically active metal electrodes including mainly Cu [30] and Ag [27]. Finally, there are some novel electrodes, such as ITO [39], FTO [42], the graphene [37], which are generally used to prepare flexible and transparent memristors, TiN [16] electrodes prepared using CMOS processes, and alloy electrodes (such as Cu-Te [36], Hf-Ta [34], Ag-Cu [35], etc.) intentionally designed to stabilize the RS behavior.

### 2.2 RS materials

The RS materials are the most important part of the memristors, and different RS materials have different resistive characteristics. RS materials can be generally classified as inorganic and organic materials. Inorganic materials (binary oxide, perovskite, 2D materials etc.) usually exhibit a more stable, faster, and robust RS behavior, while organic materials have the advantages of high flexibility, simple preparation method and low cost.

#### 2.2.1 Binary oxides

**Figure 1.**
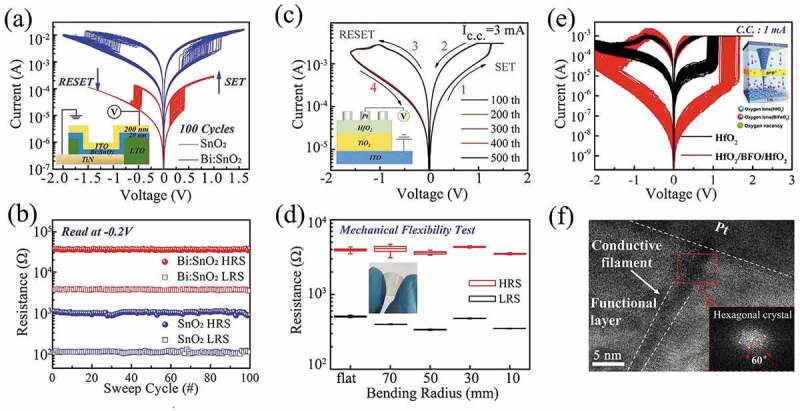
(a) I–V characteristic for ITO/SnO_2_/TiN and ITO/Bi: SnO_2_/TiN memristors. (b) The on/off state resistance ratio for ITO/SnO_2_/TiN and ITO/Bi: SnO_2_/TiN memristors. Reproduced with permission from [69], copyright 2020, Wiley. (c) Typical I–V curves of the ITO/TiO_2_/HfO_2_/Pt resistive RRAM. The inset is a schematic diagram of the device. (d) LRS/HRS resistances for the ITO/TiO_2_/HfO_2_/Pt RRAM under tensile strain with different bending radius. Inset: Photograph of the ITO/TiO_2_/HfO_2_/Pt RRAM on polyvinyl naphthalate substrate. Reproduced with permission from [49], copyright 2019, Wiley. (e) 100 sequential I–uV curves for the Pt/HfO_2_/BFO/HfO_2_/TiN and the Pt/HfO_2_/TiN devices. The inset is schematic diagram of RS mechanism of the Pt/HfO_2_/BFO/HfO_2_/TiN during the set process. (f) High-resolution TEM image of a Hf nanofilament, inserts: images of FFT patterns for hexagonal crystal Hf in the functional layer of Pt/HfO_2_/BFO/HfO_2_/TiN device. Reproduced with permission from [16], copyright 2021, Wiley.

Binary oxides have a simple composition, high stability, low cost, simple preparation process, and are compatible with the traditional CMOS process. They include TiO_x_ [46–50], SiO_x_ [51–53], AlO_x_ [54], NiO_x_ [55,56], CuO_x_ [57,58], ZnO_x_ [59,60], HfO_x_ [61,62], TaO_x_ [63,64], WO_x_ [65,66], ZrO_x_ [67,68], SnO_x_ [69] etc. In particular, HfO_x_ and TaO_x_ are the most promising binary oxides due to their sub-ns operation speed and ultimate endurance of more than 10^10^ cycles [70]. Among the many resistive materials, binary oxides are the most abundant and have excellent resistive properties, such as ultra-high on/off ratio >10^10^ in HfO_x_, sub-ns switching speeds in HfO_x_ [71], and TaO_x_ [70], and extreme endurance >10^12^ cycles in TaO_x_ [70]. However, conventional binary oxide memories generally have high power consumption or low uniformity. In order to solve these problems, our group prepared a bismuth-doped tin oxide (Bi: SnO_2_) memristor with an ITO/Bi: SnO_2_/TiN structure by magnetron sputtering. As shown in Figure 1(a), the self-compliance current, switching voltage and operating current of the Bi: SnO_2_ memristor were significantly smaller than those of the ITO/SnO_2_/TiN device. As shown in Figure 1(b), the high resistance state (HRS) and low resistance state (LRS) of Bi: SnO_2_ memristors had higher resistance values, and the operating current of Bi: SnO_2_ devices was reduced by more than an order of magnitude compared to that of SnO_2_ devices. With the content of 4.8% Bi doping, the SET operating power of doped device was 16 µW for ITO/Bi: SnO_2_/TiN memory cell of 0.4 × 0.4 µm^2^, which was cut down by two orders of magnitude. Transmission electron microscopy (TEM) observation of the Bi: SnO_2_ devices revealed that the bismuth atoms surround the surface of SnO_2_ crystals to form the coaxial Bi CFs [69].

On the other hand, with the rapid development of portable and wearable devices, it is urgent to develop flexible memristors for data storage and integration with other electronic devices in flexible electronic systems. We have fabricated a resistance random access memory (RRAM) with bilayer TiO_2_/HfO_2_ structure based on polyethylene naphthalate substrate, as illustrated in Figure 1(c), which had 500 times of mechanical bending. The coefficients of variation for the high and low resistance states were 3.2% and 3%, respectively. In Figure 1(d), no degradation of performance was observed under mechanical stresses with bending radii ranging from 70 to 10 mm. The asymmetric hourglass-like oxygen vacancy (Vo) distribution at the HfO_2_/TiO_2_ interface played a key role in the performance of this flexible RRAM device [49].

In order to improve the performance of the memristors, optimize simulate synaptic function and perform neuromorphic calculations, our research group designed a three-layer HfO_2_/BiFeO_3_(BFO)/HfO_2_ memristor by inserting a 4 nm conventional ferroelectric BFO layer. As demonstrated in Figure 1(e), the memristor has improved RS performance, such as with the storage window of 10^4^ and the multi-level storage capability. The pattern recognition simulation based on neuromorphic network was conducted with recognition accuracy of 91.2%. The CFs consisting of single crystals of hafnium (Hf) with a hexagonal lattice structure were observed using high-resolution TEM (Figure 1(f)). Attributed to the BFO inserting layer, more oxygen vacancies originate from the BFO layer, which makes the formation of Hf CF become easier [16].

Not only academics, but also the industry has engaged in the research on binary oxides. In 2010, Unity reported a 64 Mb test chip based on oxide memristor [72]. In 2013, Panasonic produced a microcontroller named MN101 L, which adopted TaO_x_-based memristor using 0.18 μm process as an embedded memory. In 2014, Sony fabricated a 16 Gb test chip based on oxide memristors using a 27 nm process. In 2016, the Institute of Microelectronics of the Chinese Academy of Sciences fabricated a 4-layer self-selective oxide memristor array with 3-dimensional vertical structure as a test chip. Taiwan Semiconductor Manufacturing Company Limited (TSMC) has been able to produce embedded oxide memristors modules using a 22 nm process [73]. Samsung reported a Pt/Ta_2_O_5_/TaO_x_/Pt memristor with a switching speed of 10 ns and an endurance of 10^12^ cycles [74]. Liu’s group in the Institute of Microelectronics of the Chinese Academy of Sciences designed a Ta/TaO_x_/W memristor with a 2-transistor 2-resistor (2T2 R) PUF scheme with assisting circuit techniques for dense and reliable cryptographic key generation [75].

#### 2.2.2 Perovskites

**Figure 2.**
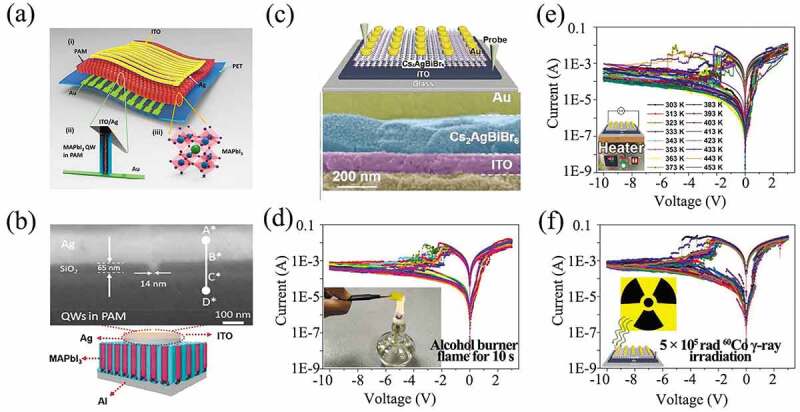
(a) (i) Schematic showing the MAPbI_3_ quantum wires (QWs)/nanowires (NWs) in a porous alumina membrane stacked between crisscrossed Ag/ITO and Au finger electrodes, supported by a polyethylene terephthalate substrate. (ii) An enlarged view of an individual QW comprising the Ag filament sandwiched between ITO/Ag and Au contacts. (iii) Crystal structure of MAPbI_3_. (b) Schematic diagram of MAPbI_3_ QWs memory device. Insert: Highly magnified cross-sectional view of 153 nm^2^ device after Ag evaporation showing the distinct SiO_2_ layer between the Ag electrode and MAPbI_3_QWs. Reproduced with permission from [17], copyright 2021, American Chemical Society. (c) Schematic diagram of the Cs_2_AgBiBr_6_-based device. I–V characteristics of Cs_2_AgBiBr_6_-based device in different harsh environments: (d) Burnt by luminous cone of alcohol lamp for 10 s. (e) Heated from 303 to 453 K. (f) Exposed under ^60^Co γ-ray irradiation with a total dose of 5 × 10^5^ rad (SI). Reproduced with permission from [94], copyright 2019, Wiley.

Perovskites are materials with ABX_3_ structure. The doping of the A-site with metal ions of low valence causes oxygen vacancies as the material [76]. By partially doping the A- and B-site with cations of different valence or radius, a wide variety of physical and chemical properties can be achieved. Where A is NH_2_CH=NH_2_^1+^ (FA^1+^), CH_3_NH_3_^1+^ (MA^1+^), or Cs+, Ag+; B is Pb^2+^, or Sn^2+^; and X is Cl-, or Br-, or I- or a combination of them [77]. Common perovskites include MAPbI_3_ [78–81], (C_4_H_9_NH_3_)_2_PbBr_4_ [82], LaFeO_3_ [83], MAPbBr_3_ [84], CH_3_NH_3_PbClXI_3_ [85–87], etc. Due to the superior optoelectronic properties of perovskite materials, such as suitable tunable band gap, high optical absorption coefficient, high photoluminescence quantum yield, narrow-band emission, relatively short absorption wavelength, long diffusion length and low cost, perovskites are ideal materials for solar cells [88,89], photodetectors [90,91], light-emitting diodes [92,93], etc. Because of novel optoelectronic properties such as mixed ion and electron conductivity, switchable majority carrier concentration and slow photocurrent decay, perovskites are promising materials for the design of next-generation neuromorphic memristors. As illustrated in Figure 2(a), Poddar et al. fabricated an RRAM with a perovskite quantum well/nanowire (NW) array as the switch matrix, with an on/off ratio of ~10^7^, multi-bit memory capability, ultrafast programming speed of ~ 100 ps (Figure 2(b)) [17].

A serious deficiency of perovskite materials is lack of environmental stability. To solve this problem, Cheng et al. firstly utilized lead-free double perovskite Cs_2_AgBiBr_6_ for environmentally robust memristors [94]. A memristor with ITO/Cs_2_AgBiBr_6_/Au structure exhibited performance of 10^5^ seconds of retention and 10^4^ times of mechanical bending (Figure 2(c)). Most importantly, the performance of the memristor remains robust in harsh environments. The device could last 10 s in alcohol burner flames, and withstand a temperature of 180°C or ^60^Co γ-ray irradiation at a dose of 5 × 10^5^ rad (SI) (Figure 2(f)). These parameters are superior to those of commercial flash memory devices [94]. Although memristors based on perovskite have been hotly investigated, the perovskite memristor still has some disadvantages like the incompatibility with CMOS processes, which limits their practical applications.

#### 2.2.3 Organic materials

With the rapid development of portable and wearable devices, it is urgent to develop flexible memristors for data storage in flexible electronic systems. Due to the low cost, solution processability, flexibility and the ability of large scale preparation, the organic materials (organic small molecules SU-8 [95], monochloro copper phthalocyanine (ClCuPc) [96], Ru-complex of 2-(phenylazso)pyridine ([Ru^II^(L)_3_] (PF_6_)_2_) [97], and organic polymers PEDOT: PSS [98,99], fluoropolymer [100], etc.) are promising candidates for flexible memristors. Liu et al. prepared an Ag/2DP_BTA+PDA_/ITO memristor, which exhibited good switching performance with high reliability and reproducibility, with switching ratios ranging from 10^2^ to 10^5^, depending on the thickness of the film [101]. However, the 2DP-based non-volatile memristor had an endurance of only 200 cycles at 0.1 V [101]. To overcome the problem of low endurance in organic memristors, Xu et al. reported a new, flexible, and robust diffusion memristor based on a copolymer of trifluorochloroethylene and vinylidene fluoride (FK-800). The device with the structure of Ag/FK-800/Pt had an switching endurance of over 10^6^ cycles [102].

**Figure 3.**
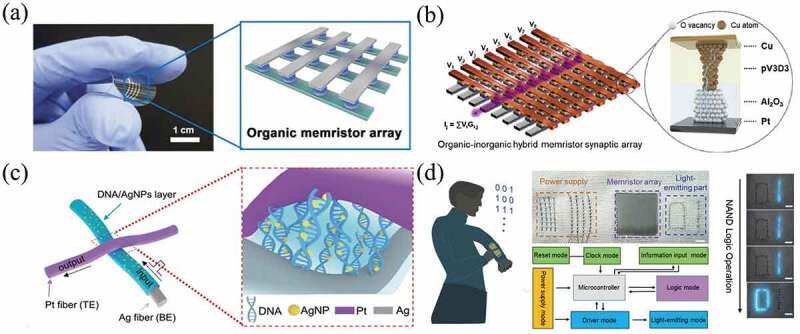
(a) Mechanical and electrical performances of the developed organic memristor. Reproduced with permission from [104], copyright 2021, Wiley. (b) Schematic illustrations of the array consisting of pV_3_D_3_/Al_2_O_3_ memristors and their multiply-accumulate operations. Reproduced with permission from [105], copyright 2022, Wiley. (c) Textile chip made from DNA-bridged memristor cross-bar arrays. Each contact point represents a memristor. (d) Photograph and the operation mechanism of a proof-of-concept all-fabric data-processing system. Scale bars in (e) is 0.5 cm. Reproduced with permission from [106], copyright 2020, Wiley.

Although the endurance of the organic memristors with the structure of Ag/FK-800/Pt has been greatly enhanced, their manufacturing yield is still low. Zhang et al. achieved a record yield of 90% for polymer memristors with miniaturization and low power consumption by implementing a 2D conjugation strategy [103]. This organic memristor had a low power consumption of 10^−15^ J/bit by constructing coplanar macromolecules with 2D conjugated thiophene derivatives [103]. Park et al. fabricated a solution-processed flexible organic memristor array with self-selectivity by systematically designing the diffusion of carbon fibers in a polymer medium, as shown in Figure 3(a). In this array, the cellular self-selectivity effectively suppressed the sneaking current paths and the maximum size of the memristor arrays is found to be more than 1 M bits. Meanwhile the neural networks based on these devices achieved a high recognition accuracy of over 90% [104].

To improve the reliability of the memristors based on organics, Cha etal. prepared Cu/poly (1,3,5-trivinyl-1,3,5-trimethylcyclotrisiloxane) (pV_3_D_3_)/Al_2_O_3_/Pt memristors, using pV_3_D_3_ and Al_2_O_3_ films for organic-inorganic bilayer stacking of synaptic unit cells [105], as illustrated in Figure 3(b). The 5-bit multilevel retention of 10^4^ s was firstly achieved. The added layer of Al_2_O_3_ played a key role in mitigating Joule heating of RESET process and conductivity fluctuations, resulting in reliable retention characteristics and stable switching behavior [105].

Some novel organic materials (deoxyribonucleic acid (DNA) [106,107] and egg proteins [108], etc.) have received widespread attention because they are biodegradable, bioabsorbable, environmentally friendly, biocompatible, implantable, and cheap [109,110]. Xu et al. prepared a robust DNA-bridged memristor based on electrophoretically DNA active layers on fiber electrodes (Figure 3(c)). The unique structure and orientation of the DNA molecule combined with silver nanoparticles provided best-in-class performance, such as a low operating voltage of 0.3 V, low power consumption of 100 pW, and high switching speed of 20 ns. As demonstrated in Figure 3(d), basic logic calculations, such as implication and NAND, were demonstrated as a function of the textile chip, and a full-fabric information processing system was demonstrated by integrating the memristor with the power and light-emitting modules [106].

#### 2.2.4 2D materials

**Figure 4.**
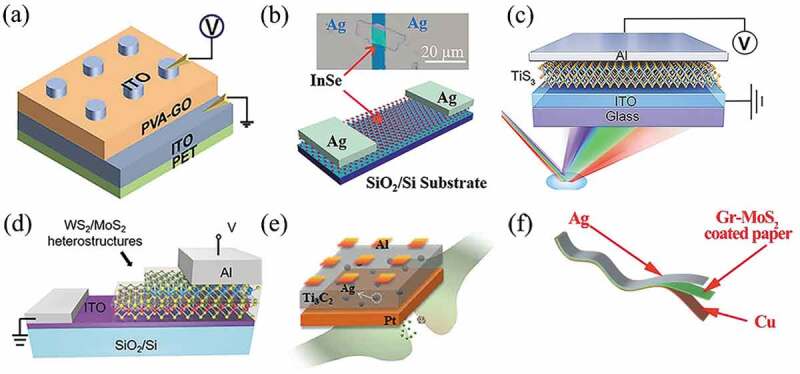
(a) Schematic of an ITO/PVA-GO/ITO memristor, where PVA stands for poly(vinyl alcohol) and GO for graphene oxide. Reproduced with permission from [112], copyright 2020, Wiley. (b) Schematic of an Ag/InSe/Ag memristor. Inset is the optical image of the device with a channel length of 8 μm. Reproduced with permission from [113], copyright 2021, AIP Publishing LLC. (c) Sandwich-like structure of the TiS_3_-based memristor. Reproduced with permission from [17], copyright 2021, American Chemical Society. (d) Schematic diagram of the WS_2_/MoS_2_ heterojunction memristor. Reproduced with permission from [111], copyright 2021, Royal Society of Chemistry. (e) The typical structure of Al/Ti_3_C_2_: Ag/Pt device. Reproduced with permission from [114], copyright 2021, Elsevier. (f) Schematics of the multilayer Gr/MoS_2_(MGM) cellulose paper memory. Reproduced with permission from [115], copyright 2019, American Chemical Society.

2D materials have attracted widespread attention due to their atomic-scale thickness as well as their excellent electrical properties and novel characteristics, such as flexibility and transparency [56]. A single layer or several layers can be exfoliated from the bulk 2D crystal due to weak van der Waals (vdW) forces between adjacent layers [111]. These characteristics make 2D materials as an alternative candidate for the atomic-scale thickness, excellent electrical properties, and novel characteristics, such as flexibility, transparency. For these reasons, researchers investigated 2D memristors with vertical and planar structures. As illustrated in Figure 4(a), our group proposed flexible memristors with vertical structures based on polyvinyl alcohol-graphene oxide hybrid nanocomposites [112]. These devices excellent optical and electrical properties (a narrow band gap (0.2 − 2 eV) and respond to light spanning from the visible to infrared regimes) with high stability against mechanical stress at voltages below 0.5 V. Tang et al. prepared an Ag/InSe/Ag memristor using 2D InSe nanosheets (Figure 4(b)) [113]. The device had a low SET voltage of 0.3 V and an on/off ratio of 4.5 × 10^3^ at a read voltage of 0.1 V. Furthermore, external strain, light, electric and magnetic fields can be applied to boost up the characteristics of 2D based memristors. Our research group designed a 2D transition metal trihalide (TMTC)-based optoelectronic memristor, as shown in Figure 4(c). The memristor exhibited stable bipolar RS property due to the excellent optical and electrical properties of titanium trisulfide (TiS_3_) functional layer. Multi-level storage was achieved by applying the wavelengths between 400 and 808 nm, and the synaptic properties such as Long-term Depression (LTD), Long-term Plasticity (LTP) and Spiking-timing-dependent plasticity (STDP) were realized. Besides, Pavlovian associative learning was successfully mimicked on the TiS_3_-based artificial synapses [40].

Owing to the small thickness of 2D material, the 2D materials based memristors normally have low endurance (tens of cycles), poor performance (on/off ratio <10), and unstable operation. Hence, vdW heterojunctions have attracted attention due to their controllable, scalable, and programmed synthesis techniques [116,117]. The vdW heterojunctions with high quality heterojunction interfaces are formed by stacking different 2D materials together by vdW forces. The heterojunctions can be constructed by selecting different 2D materials, adjusting the energy band structure, controlling the transport of the electrons, and mildly modulating the resistive properties by voltage. Since no functional layer materials are damaged during this process, the final prepared devices have good performance and endurance and can operate stably for a long time. The heterostructure of 2D materials in memristors are stacked with graphene [118,119], transition metal dichalcogenide (TMD, including MoS_2_ [116,117], WSe_2_ [120,121], MoTe_2_ [122,123], etc.), black phosphorus (BP) [124–126] and hexagonal boron nitride (h-BN) [127,128] to form vdW heterojunctions with atomically flat interfaces. Zhang et al. used a low-cost transfer-free 2D material synthesis method to fabricate a novel memristor based on WS_2_/MoS_2_ 2D semiconductor heterojunctions (Figure 4(d)) [111]. The metal/heterojunction/metal (MHM) structure utilized a reliable band modulation mechanism rather than a random ion filament formation mechanism to achieve good memristive properties. The RS mechanism based on band modulation instead of CFs could be implemented to eliminate material degradation during SET/RESET process. A prototype MHM memristor based on WS_2_/MoS_2_ heterojunction was successfully developed with a switching ratio of 10^4^ and an endurance over 120 switching cycles, showing that the performances of memristor based on 2D WS_2_/MoS_2_ heterojunction are superior to single MoS_2_ or WS_2_ layer memristors [111].

In order to improve the electrical, magnetic and optical properties of 2D materials, some researchers chased doping to modify 2D materials. Yan et al. fabricated a 2D MXene Ti_3_C_2_-based memristor (Figure 4(e)) by silver nanoparticle doping [114]. Compared with the pure Ti_3_C_2_ device, the silver nanoparticle-doped Ti_3_C_2_ memristor exhibited bidirectional continuous current transition behavior. This method not only overcome the issue of poor electrical performance of conventional memristors due to the dissolution of silver electrodes under electric fields, but also solved the problem of abrupt behavior of pure Ti_3_C_2_ devices. Meanwhile，the device realized basic decimal arithmetic operations, such as addition and multiplication [114].

Generally, 2D material-based memristors are prepared on silicon wafers or polymer substrates, but these substrates are neither cheap nor biodegradable. In contrast, cellulose paper is a good alternative due to its good biodegradability, low cost, recyclability, light weight and mechanical flexibility. As demonstrated in Figure 4(f), Yalagala et al. prepared a paper-based flexible memristor device for the first time, which used a nanohybrid material consisting of a multilayer graphite/MoS_2_ with silver and copper as the top and bottom electrodes, respectively. The memristor exhibited an endurance of 500 cycles, a switching ratio of ~10^4^, and outstanding flexibility [115].

However, 2D materials still face the following challenges: 1. Large area preparation; 2. Instability in the ambient atmosphere, which leads to performance degradation. Usually, h-BN and organic molecular films are used as vdW packaging layers, which significantly improve the device performance and its stability in the ambient atmosphere. Oxide dielectric passivation is feasible. Due to the suspended bondless surface of the 2D materials, additional seed layer growth and oxidation processes are required, but these processes may introduce impurities or defects to disrupt the inherent material structure [129]. 3. Controllable doping. Conventional doping methods such as thermal diffusion and ion implantation cause damage to atomically thin 2D materials. The type of charge carriers in 2D semiconductors strongly depends on the contacts and surrounding dielectrics, so the introduction of suitable metal contacts and dielectrics with fixed charges may be an effective strategy to achieve stable doping of 2D semiconductors. Furthermore, it is technically possible to tune the energy band structure and electronic properties of 2D materials by ion intercalation and surface modification [129]. 4. Direct deposition of metal electrodes on 2D materials usually breaks covalent bonds in the atomic lattice and introduces defects, resulting in Fermi level pinning, forming Schottky contacts instead of the desired ohmic contacts, and generating large contact resistance [129].

## 3. Structure design

**Figure 5.**
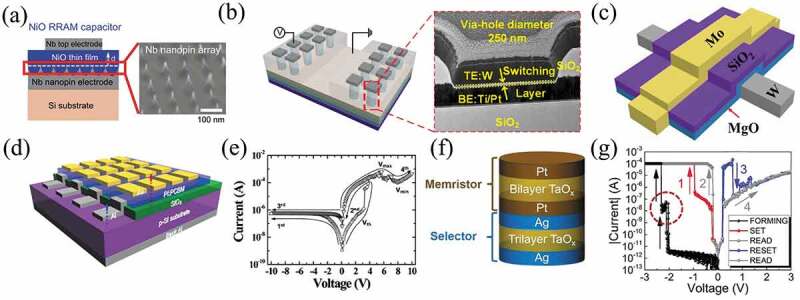
(a) Schematic drawing of a NiO RRAM cell device composed of an Nb nanopin bottom electrode, a NiO thin film, and an Nb top electrode. The thickness (d) of the NiO film is about 80 nm. Inset: A scanning electron microscopy image of the Nb nanopin array. Reproduced with permission from [20], copyright 2018, Royal Society of Chemistry. (b) Schematics of the W/AlO_x_ (RF)/Al_2_O_3_ (ALD)/Pt memristor with 250 nm via-hole structure. Reproduced with permission from [135], copyright 2020, IEEE. (c) The structure of the W/MgO/SiO_2_/Mo memristor. Reproduced with permission from [15], copyright 2021, Wiley. (d) A schematic of a 1D1R memory device. (e) IV characteristics of a 1D1R memory device. Reproduced with permission from [136], copyright 2010, Wiley. (f) 3D schematic of 1S1R integration crossbar arrays. The enlarged view is a vertical stacking cell of a selector and a memristor with top, middle, and bottom electrodes. (g) Typical I–V curves of the integrated 1S1R cell, where the forming process and normal operation cycles are included. Reproduced with permission from [137], copyright 2019, Wiley.

In order to enhance the performance of memristors, some researchers adopted electrode engineering method to modulate the performance [20,130–134]. Ahn et al. prepared Nb/NiO/Nb memristors by preparing well-aligned Nb nanopin array bottom electrodes, as shown in Figure 5(a). Due to the enhanced electric field induced by the nanopin electrode, a lower SET/RESET voltage was observed. The niobium nanopin electrode minimized the dispersion of the LRS and HRS currents and the variation of the SET/RESET voltages [20]. As illustrated in Figure 5(b), Huang et al. prepared a W/AlO_x_/Al_2_O_3_/Pt via-hole structured bilayer memristor. The devices consisted of an oxygen-deficient AlO_x_ layer and a near stoichiometric ratio Al_2_O_3_ layer which were deposited by radio frequency magnetron sputtering and atomic layer deposition, respectively. The devices exhibited high speed (28 ns), high endurance (10^8^ cycles at 100 K, 10^10^ cycles at 298 K, and 10^7^ cycles at 400 K), uniform resistance distribution, and large on/off ratio and good retention [135]. Moreover, the multilayer structure can improve the performance of the memristor [21,137–143]. Wu et al. fabricated a memristor of W/MgO/SiO_2_/Mo structure with nonvolatile analog switching characteristics, as demonstrated in Figure 5(c). The weight conductance of the memristor could be precisely adjusted and it was used to fabricate a single-layer SNN for speech recognition with a high recognition of 94%, which was equivalent to the accuracy in software [15].

**Figure 6.**
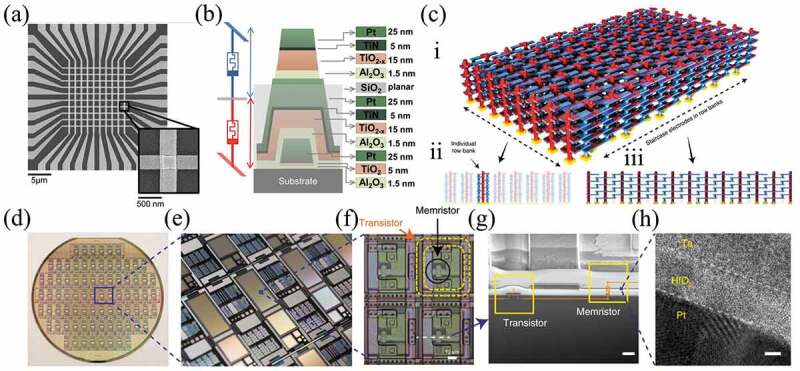
(a) Scanning electron microscopy top-view image of the fabricated circuit with a zoom on a stacked Pt/Al_2_O_3_/TiO_2–x_/TiN/Pt memristors to highlight the clean electrode edges. (b) Equivalent circuit for two Pt/Al_2_O_3_/TiO_2–x_/TiN/Pt memristors in the stacked configuration, in particular, highlighting that the middle electrode (gray) is shared between bottom (red) and top (blue) devices. The cross-section photograph showing the material layers and their corresponding thicknesses. Reproduced with permission from [155], copyright 2017, IEEE. (c) (i) 3D monolithic integrated memristor circuits. Schematic of the 3D circuits composed of high-density staircase output electrodes (blue) and pillar input electrodes (red). (ii) Sideview of 3D row banks. Each row bank in the 3D array operates independently. (iii) Sideview from column side showing unique staircase electrodes. Reproduced with permission from [22], copyright 2020, Springer Nature. (d) An optical image of a wafer with transistor arrays. (e) Close-up of chip image showing arrays of various sizes. (f) Microscope image showing the 1T1 R structureof the cell. Scale bar, 10 µm. (g) Cross-sectional scanning electron microscopic image of an individual 1T1R cell, which is cut in a focused ion beam microscope from the dashed line in (g). Scale bar, 2 µm. (h) Cross-sectional transmission electron microscopic image of the integrated Ta/HfO_2_/Pt memristor. Scale bar, 2 nm. Reproduced with permission from [141], copyright 2018, Springer Nature.

One of the advantages of memristors for next generation non-volatile memories is their good scalability. For 3D integration of memristors, the crossbar array suffers from a crosstalk current issue, which can lead to misreading of information [144]. Normally, there are four main solutions: 1. Each memory cell is connected to a diode to form a one diode one resistor (1D1 R) structure [145–147]; 2. Each memory cell at the cross-array node is connected to a transistor to form a one transistor one resistor (1T1 R) structure [141,148–151]; 3. Each memory cell is connected to a selector to form a one selector one resistor (1S1 R) structure [137,152,153]; 4. The memory cell at the cross-array node has a self-rectifying effect, that is, one resistor (1 R) structure [154]. All four solutions are designed to achieve rectification characteristics, which can suppress the interference of leakage currents. Cho et al. connected Schottky-type Al/p-Si diodes in series with an unipolar organic memories to form a 1D1 R crossover array (Figure 5(d)) [136]. Reversible switching characteristics of the unipolar memristor were observed under forward bias conditions, while under reverse bias conditions the switching was significantly suppressed due to the rectification characteristics of the diode (Figure 5(e)) [136]. As shown in Figure 5(f,g), Sun et al. developed a 1S1 R cell consisting of a Pt/Ta_2_O_5−α_/TaO_β_/Pt (PTTP) memristor and a novel Ag/TaO_x_/TaO_y_/TaO_x_/Ag (ATTA) (x<y) selector. The all-TaO_x_-based integrated 1S1 R cells were fabricated by magnetron sputtering, and exhibits nonlinear characteristics avoiding undesired crosstalk current [137]. Adam et al. reported a monolithic integrated 3D crossbar circuit based on metal oxide memristors, which was suitable for analog neuromorphic computing applications [155]. This circuit was based on Pt/Al_2_O_3_/TiO_2-x_/TiN/Pt memristors consisting of two 10 × 10 crossbars, as demonstrated in Figure 6(a,b). The integrated crosspoint memristors were optimized for analog computing applications, allowing the successful switching of 200 devices in the crossbar circuits, and most importantly, the conductivity of the device could be precisely adjusted within the dynamic operating range [155]. As shown in Figure 6(c), Lin et al. reported a 3D circuit consisting of eight layers of monolithic integrated memristor devices, which implemented a CNN function by programming parallel operation kernels into a 3D array and achieved software-comparable accuracy (98.1%) in recognizing handwritten digits in a MNIST [22]. Li et al. integrated a Ta/HfO_2_/Pt memristor with a foundry-made transistor array on a 6-inch wafer. Each memristor was connected to a series transistor in a ‘1T1 R’ configuration (Figure 6(d–h) show the integrated memristor array from the wafer scale to the nanometer scale) [141]. The device of 1T1 R structure showed robust, linear, and symmetric synaptic weight updates. As a result, a multilayer neural network implemented in a 128 × 64 memristor array was trained on the MNIST training set of 80,000 images, after which an accuracy of 91.71% was achieved on the complete 10,000 images test set [141].

## 4. Device performance

Resistive materials and device structures can decide the performances of the memristors. For non-volatile memory, we often focus on the on/off ratio [156,157], endurance [70,158], retention [159] and switching speed [160,161]. For neuromorphic applications of memristor, the conductance linearity of the device is important. In this review, the performances of memristors are introduced from the following aspects: RS performance, synaptic plasticity, and optical-electrical performance.

### 4.1 RS performance

**Figure 7.**
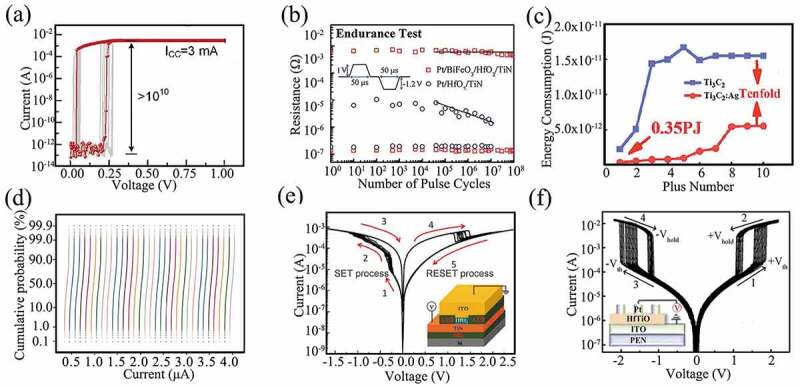
(a) Repeatable 100 cycles of Ag/TiN/HfO_x_/HfO_y_/HfO_x_/TiN/Ag device under an I_CC_ of 3 mA, indicating an extremely high ON/OFF ratio of over 10^10^. Reproduced with permission from [162], copyright 2022, Wiley. (b) Endurances of HfO_2_-based memristors before and after inserting the BFO layer. Reproduced with permission from [61], copyright 2020, Wiley. (c) The power consumption variation of Ti_3_C_2_ and Ti_3_C_2_: Ag devices. Reproduced with permission from [114], copyright 2021, Elsevier. (d) Cumulative probability distribution of 1,024 cells with respect to 32 independent conductance states. Reproduced with permission from [163], copyright 2020, Springer Nature. (e) Switching characteristics of TiN/HfO_2_/ITO device for 10 switching cycles. The inset shows a schematic diagram of the device. Reproduced with permission from [164], copyright 2014, IOP. (f) The DC I-V characteristic of the flexible Pt/Hf_17.66_Ti_13.79_O_68.55_/ITO selector device in 100 voltage sweep cycles. Reproduced with permission from [165], copyright 2019, Royal Society of Chemistry.

With a decade of research, the RS performances of the memristors have been significantly improved. The on-off ratio is defined as the ratio between the currents in the high and low resistance states of the memristor. As shown in Figure 7(a), Lu et al. fabricated a Ag/TiN/HfO_x_/HfO_y_/HfO_x_/Pt memristor, and it had an ultra-high switching ratio of 10^10^ [162]. We have fabricated a Pt/BFO/HfO_2_/TiN device by inserting a 2 nm BiFeO_3_ layer, achieving a pulse endurance of 10^8^ cycles (Figure 7(b)) [61]. Lee et al. demonstrated an TaO_x_-based memristor with an endurance of more than 10^12^ cycles [70].

Power consumption is also a key parameter in memristors, especially in order to solve the power wall problem [166]. Wang et al. fabricated 2D MXene Ti_3_C_2_-based memristors and the power consumption was 0.35 pJ/bit, as illustrated in Figure 7(c) [114]. Fu et al. fabricated a forming-free V/VO_x_/HfWO_x_/Pt memristor and power consumptions for writing and erasing are 0.06 pJ/bit and 0.9 pJ/bit, respectively [26]. Wang et al. proposed an ultra-low power Ta_2_O_5_/Al_2_O_3_ bilayer-gate-dielectric synaptic transistor and it showed a low power consumption of 19.9 aJ/bit [167].

Multi-level property may be concerned for memristors. Yao et al. implemented CNNs based on high-yield, high-performance and uniform memristor crossbar arrays. The CNNs integrated eight 2,048-cell memristor arrays to improve parallel-computing efficiency [163]. Figure 7(d) shows the distribution of 1024 memristors with 32 different conductance states, where all curves are separated without any overlap. This system with a five-layer memristor-based CNN achieved a high accuracy of over 96% to perform MNIST image recognition [163].

The switching speed reflects the ability of the memory to achieve fast operation, and the high switching speed shows the great potential of the memristors as high-speed memory. Poddar et al. developed a memristor with a high density of monocrystalline perovskite quantum wires as the switching matrix, showing a switching ratio of ~10^7^ and an ultrafast switching speed of ~ 100 ps [17]. Besides the above properties, self-compliance of current has been reported, which is used to simplify the peripheral circuits. As demonstrated in Figure 7(e), our research group fabricated a memristor with TiN/ITO structure, which demonstrated a self-compliance current effect for the ITO electrode [164]. Another novel characteristic of memristor is self-rectifying effect，which can effectively suppress the crosstalk current and significantly simplify the complexity of circuit [168–175]. Besides, selector is often combined with memristor for 3D integration. As illustrated in Figure 7(f), we fabricated a Pt/HfTiO/ITO selector on a flexible substrate polyvinyl naphthalate (PEN), which exhibited excellent bending reliability in the bending radius range of 50 mm to 30 mm without degradation of the operating performance. This selector device could be switched on within 60 ns at 4.4 V and turned off within 50 ns under a 0.5 V bias [165].

Commercial non-volatile memories must meet requirements for integration with current ICs. Among these requirements are integration density up to 1 gigabyte (GB)/mm^2^, writing voltage <3 V, power consumption <10 pJ, switching speed <10 ns, endurance >10^10^ cycles, HRS/LRS resistance ratio >10, and low resistance fluctuation over time if no bias is applied (preferably less than 10% over >10 years) [176]. As shown in Table 1, which presents a comparison of the Set/Reset voltage, retention, switching ratio, endurance, operation speed for different types of memristors. For the oxide based device, the memristor with the structure of Ag/TiN/HfO_x_/HfO_y_/HfO_x_/Pt has the ultra-high on/off ratio >10^10^ and sub-ns switching speeds [71], but it has some disadvantages, such as high power consumption or low uniformity. In order to solve the problem, doping in the switching layer [177], design double layer structure [178,179] have been taken by some academics. For Perovskites based device, it can be seen that the memristor with the structure of ITO/Ag/MAPbI_3_/Au has the operation speed of 100 ps and the on/off ratio of 10^7^ [17]. But the environmental stability needs to be improved. Some scholars have improved the environmental stability of the memristors based on perovskites by new materials of functional layers, such as lead-free bicalcitonite Cs_2_AgBiBr_6_ [94]. For organic memristors, the memristor with the structure of Ag/SU-8_Ag_/Pt has low power consumption of 1.5 fW and the switch ratio of 10^6^ [95]. To improve the endurance of the memristors based on organics, organic-inorganic bilayer stacking has been taken [105]. For 2D based memristors, low endurance (tens of cycles), poor performance (on/off ratio <10) are obstacles to the future applications. The preparation technology of the functional layer is important. It can be seen that the memristor with the structure of Pt/h-BN/Ag has the low Set/Reset voltage of 0.3/−0.1 V, switch ratio of 10^8^, endurance of 10^7^ cycles and operation speed of 50 ns [127].

**Table 1.** Comparison of different types of memristors.

Configuration	Structure	Set voltage (V)	Reset voltage (V)	Retention (s)	Switching ratio	Endurance (cycles)	Operation speed	Ref.
Oxide-RRAM	Pt/Ta_2_O_5-x_/TaO_2-x_/Pt	−1	2	–	>10	10^12^	10 ns	[70]
	Ag/TiN/HfO_x_/HfO_y_/HfO_x_/Pt	–	–	–	10^10^	10^6^	60 ns	[71]
	Pt/SiO_x_:Pt/Ta	~-0.6	1–1.2	10^7^	10^3^	3 × 10^7^	<100 ps	[53]
Perovskite-RRAM	ITO/Ag/MAPbI_3_/Au	2.4	−2.2	4.2 × 10^7^	~10^7^	~10^6^	100 ps	[17]
	ITO/Cs_2_AgBiBr_6_/Au	1.53	−3.4	10^5^	>10^2^	10^3^	-	[94]
Organic-RRAM	Ag/SU-8_Ag_/Pt	<0.3	<0.7	2 × 10^3^	~10^6^	10^2^	-	[95]
	Ag/2DP_BTA+PDA_/ITO	0.90	−1	8 × 10^4^	~10^5^	2 × 10^2^	-	[101]
	Ag/FK-800/Pt	–	–	–	>10^2^	>10^6^	340 μs	[102]
	Ag/DNA/AgNP/Pt	0.3	−0.2	10^5^	10^6^	1000	20 ns	[106]
2D-RRAM	ITO/PVA-GO/ITO	−0.2	0.2	10^4^	>10	5 × 10^2^	-	[112]
	Ag/InSe/Ag	0.3	−0.7	3.5 × 10^4^	4.5 × 10^3^	3 × 10^2^	-	[113]
	Pt/h-BN/Ag	0.3	−0.1	–	10^8^	10^7^	50 ns	[127]

### 4.2 Synaptic plasticity

**Figure 8.**
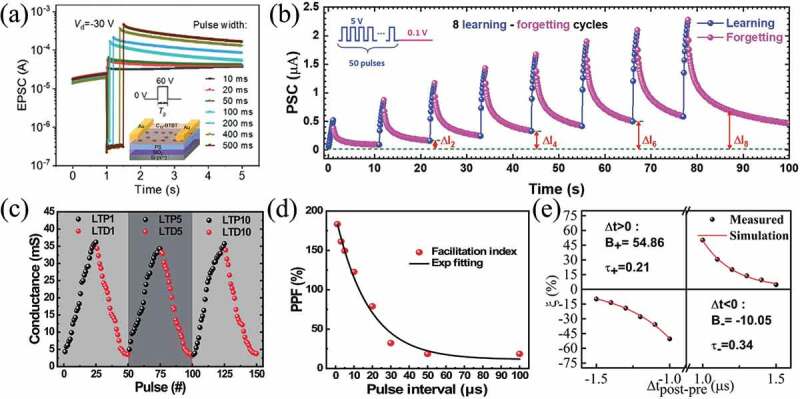
(a) EPSC triggered by positive gate voltage pulses with different widths. Insert: schematic of organic field-effect transistor [191]. (b) The learning experience behaviors of Cu/SiC/W memristor for the learning/forgetting process [192]. (c) The repeated LTP and LTD characteristics of the device demonstrating good stability with minimal cycle-to cycle variations. (d) PPF index obtained by applying paired pulses [193]. (e) Asymmetric Hebbian STDP rule obtained in HfO_2_/BFO/HfO_2_ memristor [16].

Memristors have become an ideal candidate for artificial synaptic devices in neuromorphic systems. To simulate synapses, the memristor exhibit a simulated switching behavior that requires a gradual SET and RESET processes rather than an abrupt one [180]. Recently, some research groups have investigated synaptic properties using materials based on ion migration or electrochemical reduction reactions, such as TiO_x_ [181], HfO_x_ [182], TaO_x_ [183,184], ZnO_x_ [57,60], organic [185–187] et al. Qiao et al prepared a memristor base onC_12_-BTBT. As shown in Figure 8(a), a positive voltage pulse with the assistance of UV light irradiation (1.35 mW cm^−2^) was applied to the memristor to trigger the excitatory postsynaptic currents (EPSC) [188]. Kapur et al. fabricated a memristor based on silicon carbide (SiC) and the conductance could be modulated gradually. Moreover, the synaptic function of learning-forgetting-relearning processe was successfully emulated and demonstrated using a 3 × 3 array of SiC-based memristors (Figure 8(b)) [189]. Ali et al. fabricated memristors with Ag/GeSe/Pt/Ti/SiO_2_ structure and simulated synaptic functions, such as long time range enhancement (LTP), long time range inhibition (LTD) (Figure 8(c)), paired pulse facilitation (PPF) (Figure 8(d)) and others [190]. Our group prepared a Pt/HfO_2_/BFO/HfO_2_/TiN artificial synapse, realizing the physiological synapse STDP characteristics (Figure 8(e)) [16]. Furthermore, the memristors simulated other synaptic functions, such as short-term potentiation, post-tetanic potentiation, nonassociative learning, associative learning, synaptic scaling and spike-rate-dependent plasticity [189].

### 4.3 Optical-electrical performance

**Figure 9.**
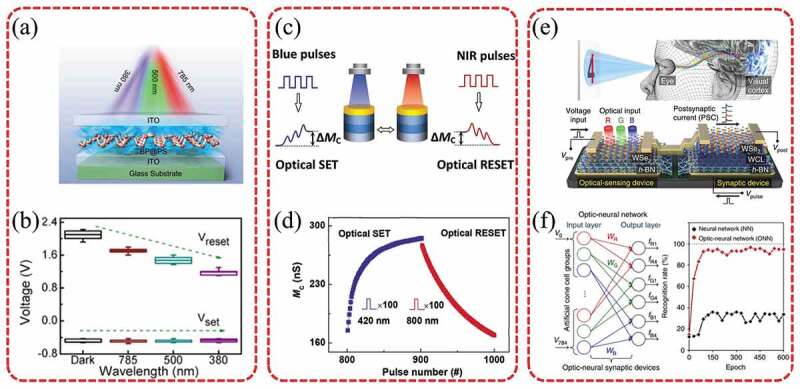
(a) Schematic showing light modulation of the BP@PS memristor. (b) I-V curves of the BP@PS memristor modulated by different wavelengths. Reproduced with permission from [23], copyright 2020, American Chemical Society. (c) Schematic of the realization of an AOC memristor. MC and NIR denote the memconductance and near-infrared, respectively. (d) Reversible regulation of the memconductance by means of 100 blue light pulses (D = 1 s, I = 1 s, and *P* = 20 µw cm^−2^) and 100 NIR light pulses (D = 1 s, I = 1 s, and *P* = 24 µw cm^−2^). Reproduced with permission from [191], copyright 2021, Wiley. (e) Integration of the h-BN/WSe_2_ optic-neural synaptic device. A Schematic of the human optic nerve system, the h-BN/WSe_2_ synaptic device integrated with h-BN/WSe_2_ photodetector, and the simplified electrical circuit for the ONS device. Here, the light sources were dot lasers with wavelengths of 655 nm (red), 532 nm (green), and 405 nm (blue) with a fixed power density (P) of 6 mW cm^−2^ for all wavelengths. (f) Left: Developed ONN for recognition of 28 × 28 RGB-colored images. Right: Recognition rate as a function of number of training epochs for ONN and conventional NN. Reproduced with permission from [192], copyright 2018, copyright 2021, Springer Nature.

Nowadays, photoelectric memristors attracted much attention for the excellent optical-electrical performance. As shown in Figure 9(a), Zhou et al. from our group prepared a optoelectronic memristor based on BP nanosheets (BP@PS NSs) coated with polystyrene (PS) [23]. With the aid of PS, the BP@PS-based memristor has good RS characteristics such as no initial preforming, low operating voltage, and long retention time. As demonstrated in Figure 9(b), during illumination ranging from ultraviolet (380 nm) to near infrared (785 nm), the Schottky barrier height is elevated further so that the resetting voltages and power consumption decrease [23]. The conductance of most optoelectronic synaptic devices could only be reversibly tuned by a combination of optical and electrical signals [193–195]. For an ideal optoelectronic neuromorphic device, its weight is represented by the conductance, which should be all optically tunable. Hu et al. implemented an all-optical control analog memristor based on InGaZnO switching layer (Figure 9(c)) [191]. As illustrated in Figure 9(d), the membrane conductance of this memristor was reversibly tunable in a continuous range only by changing the wavelength of the incident light. The photoinduced membrane conductance state was non-volatile. This device simulated STDP by all-optical modulation [191]. Moreover, some scholars have applied memristors with good opto-electronic properties for the neural network of the next generation. Seo et al. demonstrated an optical neurosynaptic device by implementing both synaptic and optical sensing functions on h-BN/WSe_2_ heterogeneous structures (Figure 9(e)) [192]. The device simulated the human visual system to recognize color and color mixed patterns in an optical neural network (ONN). The system achieved a recognition rate of over 90% for color pattern recognition tasks by this ONN (Figure 9(f)) [192]. Some academics have focused on piezoelectrical and thermoelectrical performance of memristors. Liu et al. proposed a multilevel memristor based on bamboo-like GaN microwires. The piezotronic effect was introduced to modulate the SET voltage and the general performance of the memristor [177]. Lappalainen et al. fabricated a thermal-electric VO_2_ memristor. A basic thermoelectric logic circuit of the memristor was demonstrated to be used to simulate the operation of synapses between neurons [178].

## 5. Applications

In-memory computing based memristors are normally divided into logic operations using abrupt memristors, and analog calculations using gradual memristors. The abrupt memristor has an abrupt transition between high and low resistance states, which is used as the logic input and output to realize non-volatile information processing. Gradual memristor can continuously change its resistance during SET and RESET processes, which is suitable for building efficient ANN [14]. The cycle-to-cycle variation of the memristors [179] and linearity [196] are critical factors to build high computational accuracy for ANN. In order to obtain the gradual resistances of memristors, the following methods can be used to fabricate the memristor such as doping in the switching layer [197], design double layer structure [198,199], electrode engineering [200], etc.

### 5.1 Logic operation

**Figure 10.**
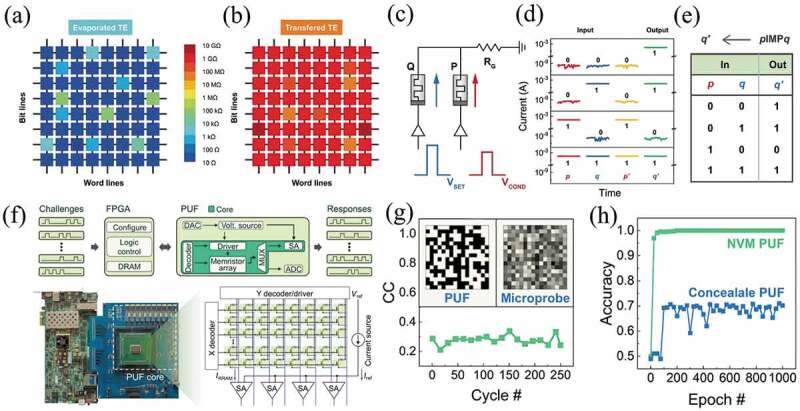
(a) Au/h-BN/Au memristors array and IMP logic implementation. The color map plots of the resistance distribution of the 8 × 8 memory array fabricated via (a) Direct metal evaporation and (b) Metal transfer technique. (c) Schematic of the IMP logic circuit. V_SET_ and V_COND_ are simultaneously applied to memristors Q and P, respectively. RG is chosen to be 500 Ω. (d) Experimental results of IMP operations for four input conditions. p and q are the resistance states of device P and Q, respectively. “1” refers to low-resistance state and “0” refers to high-resistance state. p′ and q′ are the states of device P and Q, respectively after the logic operations. Variable p remains unchanged because V_COND_ is lower than V_SET_, so p and p′ should have the same value. The readout voltage is 0.1 V. (e) True table of the logic operation. Reproduced with permission from [202], copyright 2022, Wiley. (f) Photograph of the PUF system. ZC706 FPGA evaluation board from Xilinx is used. The chip contains an 8-kb memristor array and supports parallel operation for up to eight memristor devices. (g) The change in CC between binary PUF data (top left inset) and analog resistance values measured in secure mode (top right inset). (h) The change of prediction accuracy with incremental training epoch for traditional NVM PUF, whose data are steadily stored by distinct high and low conductance states, and the developed concealable PUF. Reproduced with permission from [203], copyright 2022, American Association for the Advancement of Science.

The conventional CMOS digital logic operations are usually based on complementary transistors. This transistor-based logic circuit cannot maintain the logic state after power failure. Digital logic operations based on non-volatile memristors can solve this problem, because its logic output is the resistance of the device. The memristors array in the chip can perform distributed parallel computing, which will greatly improve the computational efficiency [201]. Mao et al. reported a damage-free Au/h-BN/Au memristor using a clean, water assisted metal transfer approach by physically assembling Au electrodes onto the layered h-BN. As shown in Figure 10(a,b), the memory arrays fabricated by vdW metal integration technology and direct metal evaporation. By using the memristors as logic gates and latches, simultaneous data storage and ‘stateful’ implication logic (IMP) is achieved in a longitudinal array with suppressed potential path currents (Figure 10(c–e)) [202]. Krishnaprasad et al. demonstrated low variability synapses using chemical vapor deposited 2D MoS_2_ as a switching medium with Ti/Au electrodes [204]. Logical operations of AND, OR, and NOT were implemented by monolithic integration of MoS_2_ synapses with MoS_2_ leaky integrate-and-fire neurons [204]. Yuan et al. demonstrated an efficient logic method based on Pt/Ta/Ta_2_O_5_/Pt/Ti memristors, where the two input variables were represented by different physical quantities, implementing all 16 Boolean logic functions in less than 3 steps using single device [205].

With the advent of the era of big data, the security of personal information and hardware is becoming more and more important. The physical unclonable function (PUF) is a promising security primitive that uses the random variations inherent in electronic hardware to generate digital keys [206]. As demonstrated in Figure 10(f), Gao et al. certified a hideable PUF at the chip level by integrating an array of memristors [203]. The switching characteristics of HfO_x_-based memristors were used to efficiently implement PUF hiding/recovery via SET/RESET operations. A PUF recovery with zero-bit error rate and significant attack resistance was achieved. This hideable feature, coupled with the inherent noise in the memristor array, allowed the PUF to effectively resist both invasive and noninvasive attacks which were the main threats to modern hardware security. As illustrated in Figure 10(g), an attacker executes an attack in a simulated manner by analyzing the correlation between valid PUF data and conductance distribution in secure mode. In contrast, the conductance obtained from the secure mode via microprobes was disordered and had a correlation coefficient of less than 0.4 over 250 cycles. Multilayer fully connected perceptron could be used to perform such attacks on the concealable PUFs, as shown in Figure 10(h), where the trained neural network predicted the accuracy of 70% from the recovered PUF data, which is insufficient for breaking the PUF [203]. Yang et al. demonstrated the subthreshold slope variation of a transistor could be used as the entropy source of a PUF to generate a physical key [207]. By combining this subthreshold slope PUF with a memristor-based XOR logic function, an in situ encryption/decryption scheme in a compact 1T1 R structure was proposed. Experiments demonstrated that the subthreshold slope PUF had good reproducibility, uniqueness and uniformity. Encryption and decryption of three 16-bit binary sequences were successfully implemented in a 1T1 R device using a PUF key of subthreshold slope [207].

Encryption and decryption of information is another way to ensure information security. Song et al. proposed an optimized XOR logic gate based on TiN/Ti/HfO_x_/TiN memristor [208]. The encryption and decryption based on the XOR circuit consisting of two memristors was verified by successfully performing parallel electrical tests [208]. Furthermore, the true random number generator (TRNG) is critical for cryptographic applications. The advantages of TRNG based on memristors are rich and easily extractable randomness, low cost, high integration density, low operational power consumption, and fast response time [209,210]. Predictable random number generators (RNGs) open the door to the attacks that can hack the devices and compromise the data. TRNG must be unpredictable, statistically independent, and uniformly distributed. Kim et al. generated real random numbers using the random oscillation behavior of NbO_x_-mott memristors, which exhibited self-clocking, fast, and variable tolerance [211]. The random number generation rate of the device could be at least 40 kb/s which was the fastest record compared to previous volatile memristor-based TRNG devices [211].

### 5.2 Analog calculations

Analog calculations can be divided into the following two aspects: 1. Artificial sensory system. To mimicking biological sensing systems, sensor devices are further integrated with the memristor storage and computing unit, integrating sensing, computing, storage to build a sensing-memory-computing integrated processing unit [212]. 2. Neuromorphic brain-like computing. The conductance changes in most memristors originate from ionic motion, which are very similar to the processes involving neurons and synapses in the brain. Therefore, memristor is an important component for building artificial neural networks for future applications such as computer vision [126,192,194,213–215], speech recognition [216,217], driverless vehicles [218], robotics [219], finance [220], etc.

**Figure 11.**
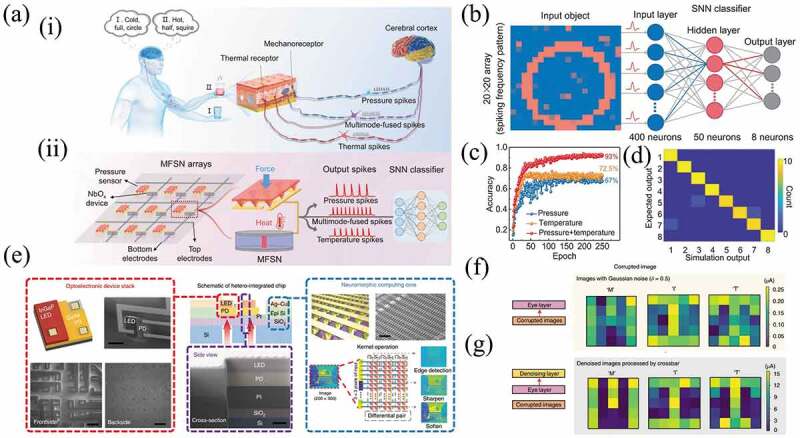
(a) (i) Artificial somatosensory system consisting of an MFSN array and an SNN classifier to emulate tactile perception. (ii) the MFSN unit is composed of a pressure sensor and a NbO_x_-based memristor. The output frequency of the MFSN unit is affected by the pressure intensity and temperature, while the output amplitude of spikes depends only on the temperature. (b) Schematic of an SNN comprising 400 input neurons, 50 hidden neurons, and 8 output neurons for the simulation. (c) Evolution of the training accuracy with training epoch at three different modes (pressure, temperature, and multimodal mode). (d) Confusion matrix of simulation classification output versus the expected outputs. The result shows the good classification ability of the MFSN-based system. Reproduced with permission from [212], copyright 2022, Wiley. (e) Three different types of 3 × 3 kernel operation (edge detection, sharpen and soften) are performed. (f) Corrupted letter images are generated by adding Gaussian noise (*δ* = 0.5) to images from the eye layer. (g) Letter images are denoised by inserting a denoising layer that includes memristor crossbar arrays, as described in the block diagram. Reproduced with permission from [221], copyright 2022, Springer Nature.

As shown in Figure 11(a), Zhu et al. reported an artificial multimodal sensory system consisting of an array of multimodal fusion spiking neurons (MFSN) operating in the spiking domain and an SNN classifier for processing multimodal sensory input without losing unimodal information (Figure 11(b)). This MFSN perceived multimodal sensory information and converted it to spikes without using a conversion module, reducing the complexity of circuit and power consumption. By feeding the spikes into the SNN classifier, the results shown that patterns with multiple modes endowed a higher recognition rate (93%) to identifying cup features than only temperature (72.5%) or pressure (67%) mode, as demonstrated in Figure 11(c). The confusion matrix (Figure 11(d)) shown the simulated classification output compared to the expected output (the color bars show the counts of the output neurons), demonstrating the excellent classification capability of the MFSN-based system [212].

Besides, memristors are expected to simulate the reconstruction of biological neural networks and realize neuromorphic brain-like computing. Choi et al. reported stackable heterogenous integrated chips, which used optoelectronic device arrays for chip-to-chip communication and neuromorphic cores based on memristor crossbar array for highly parallel data processing. They created a system with stackable and replaceable chips and successfully executed three 3 × 3 core operations (vertical edge detection, sharpening and softening) for image processing (Figure 11(e)). To test the denoising ability of the system, the authors generated corrupted letter images by adding Gaussian noise (δ = 0.5) to the image from the eye layer (Figure 11(f)). As shown in Figure 11(g), the letter images were then denoised by inserting a denoising layer including a memristor crossbar array. The results showed that the stackable and replaceable chips exhibited excellent immunity to high noise level [221]. Zhong et al. reported a dynamic memristor-based parallel reservoir computing system. The system achieved a low word error rate of 0.4% in speech digit recognition and a low normalized root-mean-square error of 0.046 in time series prediction of Hénon graphs. This result outperformed most existing hardware-based reservoir computing systems and software-based reservoir computing systems for the Hénon graph prediction task [217].

**Figure 12.**
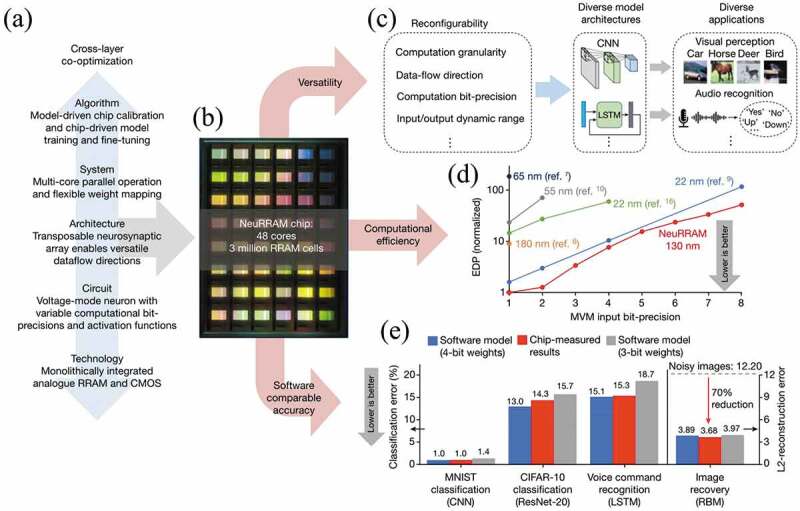
Design methodology and main features of the NeuRRAM chip. (a) Cross-layer co-optimizations across the full stack of the design enable NeuRRAM to simultaneously deliver high versatility, computational efficiency and software-comparable inference accuracy. (b) Micrograph of the NeuRRAM chip. (c) Reconfigurability in various aspects of the design enables NeuRRAM to implement diverse AI models for a wide variety of applications. (d) Comparison of EDP, a commonly used energy-efficiency and performance metric among recent RRAM-based CIM hardware. (e) Fully hardware-measured inference accuracy on NeuRRAM is comparable to software models quantized to 4-bit weights across various AI benchmarks. Reproduced with permission from [226], copyright 2022, Springer Nature.

Liang et al. developed a hardware prototype for near-sensor computing, chaotic time-series prediction and handwriting classification [222]. Zhong et al. reported a fully analogue reservoir computing system which can efficiently process spatiotemporal signals in real time [223]. For a RRAM compute-in-memory (CIM) chip to be widely adopted in real-world AI applications, it needs to simultaneously provide high energy efficiency, flexibility to support different AI model architectures and software-comparable inference accuracy. Hitherto, there has not been a study aimed at simultaneously improving all these three aspects of a design [224,225]. Through collaborative optimization from algorithms and architectures to circuits and devices (Figure 12(a)), as show in Figure 12(b), Wan et al. reported a NeuRRAM – a RRAM-based CIM chip [226]. The chips with reconfiguring CIM cores were compatible with different model architectures and their energy efficiency was two-times better than previous state-of-the-art RRAM-CIM chips (Figure 12(c,d)). Fully hardware-measured inference accuracy on NeuRRAM was comparable to software models quantized to 4-bit weights across a variety of AI tasks (Figure 12(e)), including accuracy of 99% on MNIST and 85.7% on CIFAR-10 image classification, 84.7% on Google speech command recognition [226].

## 6. Challenges and prospects

This review discussed various RS materials for memristors, including electrodes, binary oxides, perovskite, organics, and 2D materials. Besides, the role in the memristor were systematically discussed. The construction of the shaped electrode, the design of functional layer and other factors influencing the device performance are analyzed. The excellent and advanced performances of the memristor from the frontier researches are illustrated. The applications of memristors in logic and analog are presented. The prospects and challenges of memristors are highlighted, providing further developments and new insights into in-memory computing based on memristors. Despite decades of intensive research, memristors have been greatly developed. However, on the road to commercialization of memristors, there are still many issues to be solved.

Even for the same material with sandwich structure, different research groups may propose different resistive mechanisms, and this phenomenon may be caused by differences in the manufacturing process. More systematic work is needed to elucidate the resistive mechanism and failure mechanism of memristors in the future. Currently, except for HfO_x_ and TaO_x_, the vast majority of RS materials are not yet compatible with CMOS processes, which limits the practical applications. Therefore, the integration technology should support conventional CMOS compatible materials and CMOS-incompatible materials for a wider range of applications in the future.

The conductance linearity, power consumption, device-to-device (spatial) and cycle-to-cycle (temporal) characteristics of most memristors are not ideal, so device performances are needed to be further boost up. In addition, the human sensory system is a fusion of multiple senses. Therefore, developing device systems with multi-sensory fusion and diverse processing capabilities is a promising direction for future applications. As for system-level optimization, dedicated algorithms and peripheral circuits for memristor-based neuromorphic computing should be developed in order to fully exploit the performance of particular devices.

In the future, brain-like applications based on memristors need to be further expanded, such as the use of bionic brain-like computing devices to build brain-computer interface, which can provide new research ideas for medical electronics, biomedicine, and other fields. Based on the functional characteristics of the biosensory nervous system, an efficient and intelligent information perception system with biological reality can be established to create smarter bio-inspired robots.
